# Enrichment and Proteomic Characterization of the Cyst Wall from *In Vitro* Toxoplasma gondii Cysts

**DOI:** 10.1128/mBio.00469-19

**Published:** 2019-04-30

**Authors:** Vincent Tu, Joshua Mayoral, Tatsuki Sugi, Tadakimi Tomita, Bing Han, Yan Fen Ma, Louis M. Weiss

**Affiliations:** a Department of Pathology, Albert Einstein College of Medicine, Bronx, New York, USA; b Department of Medicine, Albert Einstein College of Medicine, Bronx, New York, USA; c IVD Development Unit, Medical & Biological Laboratories Co. Ltd., Ina, Nagano, Japan; University of Arizona

**Keywords:** bradyzoites, CST1, cyst wall, latency, proteomics, Toxoplasma gondii

## Abstract

The tissue cyst of Toxoplasma gondii, found in latent infection, serves a critical role in both transmission and reactivation of this organism. Within infected cells, slowly replicating parasites (bradyzoites) are surrounded by a cyst matrix, cyst wall, and cyst membrane. The cyst wall is clearly delineated by ultrastructural analysis; however, the composition and function of this layer in host-parasite interactions are not fully understood. In order to understand the composition of the cyst wall, a proteomic analysis of purified cyst wall fragments, that were enriched with Percoll gradients and subsequently immunoprecipitated with CST1 antibody, was performed. Known cyst wall proteins, such as CST1, BPK1, MCP4, MAG1, GRA2, GRA3, and GRA5, were identified in this preparation by liquid chromatography-tandem mass spectrometry (LC-MS/MS). In addition, dense granule proteins (GRAs) not previously shown to associate with the cyst wall, as well as uncharacterized hypothetical proteins, were identified in this cyst wall preparation. Several of these hypothetical cyst wall (CST) proteins were epitope tagged, and immunofluorescence assays confirmed their localization as novel cyst matrix and cyst wall proteins. Expression of two of these newly identified cyst wall proteins was eliminated by gene knockout (CST2-KO and CST3-KO). CST2-KO parasites were highly attenuated in virulence and did not establish detectable cyst burdens. This targeted proteomic approach allowed the identification of new components of the cyst wall that probably have roles in the parasite/host interface.

## Abstract (precis)

Toxoplasma gondii is a highly prevalent parasite worldwide that presents life-threatening risks to immunocompromised and pregnant individuals. Whereas the life stage responsible for acute infection can be treated, the life stage responsible for chronic infection is refractory to currently available therapeutics. Little is known about the protein composition of the cyst wall, an amorphous structure formed by parasites that is suspected to facilitate persistence within muscle and nervous tissue during chronic (latent) infection. By implementing a refined approach to selectively purify cyst wall fragments, we identified several known and novel cyst wall proteins from our sample preparations. We confirmed the localizations of several proteins from this data set and identified one that is involved in parasite virulence. These data will propel further studies on cyst wall structure and function, leading to therapeutic strategies that can eliminate the chronic infection stage.

## INTRODUCTION

Serosurvey reports suggest that 10% to 70% of a nation’s population are infected with Toxoplasma gondii (1). The latent infection stage, termed the bradyzoite, found within tissue cysts, is a critical life stage for transmission of the disease, as infection with *Toxoplasma* can occur from ingestion of latently infected tissues from animals used for meat production (2). When human individuals are infected with T. gondii, the parasites establish a persistent infection which evades host immune clearance. If infected individuals, such as individuals with advanced AIDS or patients who undergo organ transplantation, followed by immunosuppressive therapy, become immunocompromised, bradyzoites predominantly present within the CNS can reactivate and convert into the quickly replicating tachyzoite stage, leading to encephalitis (3).

A prominent characteristic of bradyzoites is that they are surrounded by a thick cyst wall beneath a cyst membrane, the interface between the cyst and infected host cell. Ultrastructural investigation of bradyzoite cysts demonstrates invaginations and a sponge-like structure underneath the parasitophorous vacuole membrane (4). Protein components of the cyst wall that have been identified include GRA2 (5), GRA5 (5), MCP4 (6), BPK1 (6), and CST1 (7). The cyst wall is hypothesized to be involved in host-parasite interactions and tolerance to environmental stress. In accordance with these hypotheses, genetic deletion of the BPK1 gene affected the transmission of the parasite through the GI tract (8), and genetic deletion of the CST1 gene reduced cyst sturdiness and cyst number in the brains of mice with persistent infection (7). However, all of the specific functions the cyst wall serves are not yet fully understood, including how this structure is formed and which proteins are related to its structural and functional formation.

Several strategies to identify proteins in the cyst wall have been reported. Hybridoma library screening for cyst wall-reactive monoclonal antibodies (MAb) led to the identification of the cyst wall protein CST1 (7); however, this strategy is restricted by the immunogenicity of cyst wall proteins. Transcriptomic analysis of genes upregulated in bradyzoite stages and predicted to contain a signal peptide has been used to discover cyst wall proteins and led to the identification of MCP4 and BPK1 (6); however, this strategy also has limitations, as signal peptide prediction and bradyzoite transcript abundance may not predict all of the bradyzoite secreted proteins that localize to the cyst wall. To develop a nonbiased proteomic identification of cyst wall components, sample preparation of the cyst wall is a crucial initial step. Zhang et al. previously succeeded in an enrichment of cyst wall fractions by affinity separation of membrane fragments using the Dolichos biflorus agglutinin (DBA) lectin, which is widely used to label the cyst wall (7), and confirmed that the antigens in the enriched fraction could raise reactive antibodies to the cyst wall (9). However, contaminating proteins from the parasite and DBA binding host cell proteins prevented this approach from providing a robust platform for proteomic identification of cyst wall proteins. In the tachyzoite stage, intracellular parasites reside inside a parasitophorous vacuole membrane (PVM), and PVM fractions have been successfully separated from tachyzoites with differential centrifugation and immune-affinity separation (10), similar to the strategy used in organelle isolation of eukaryotic cells (11). Therefore, we took advantage of Percoll density-based fractionation of fragmented infected host cells followed by immune separation of the cyst wall-containing fraction with a CST1-specific monoclonal antibody to enrich for cyst wall-containing samples and to reduce parasite body contamination. Subsequently, we conducted proteomic identification of the proteins in the cyst wall enriched fraction to provide a data set of potential T. gondii cyst wall proteins, identifying known cyst wall proteins and validating several novel cyst wall proteins in the process.

## RESULTS

### Cyst wall fraction enrichment.

**FIG 1:**
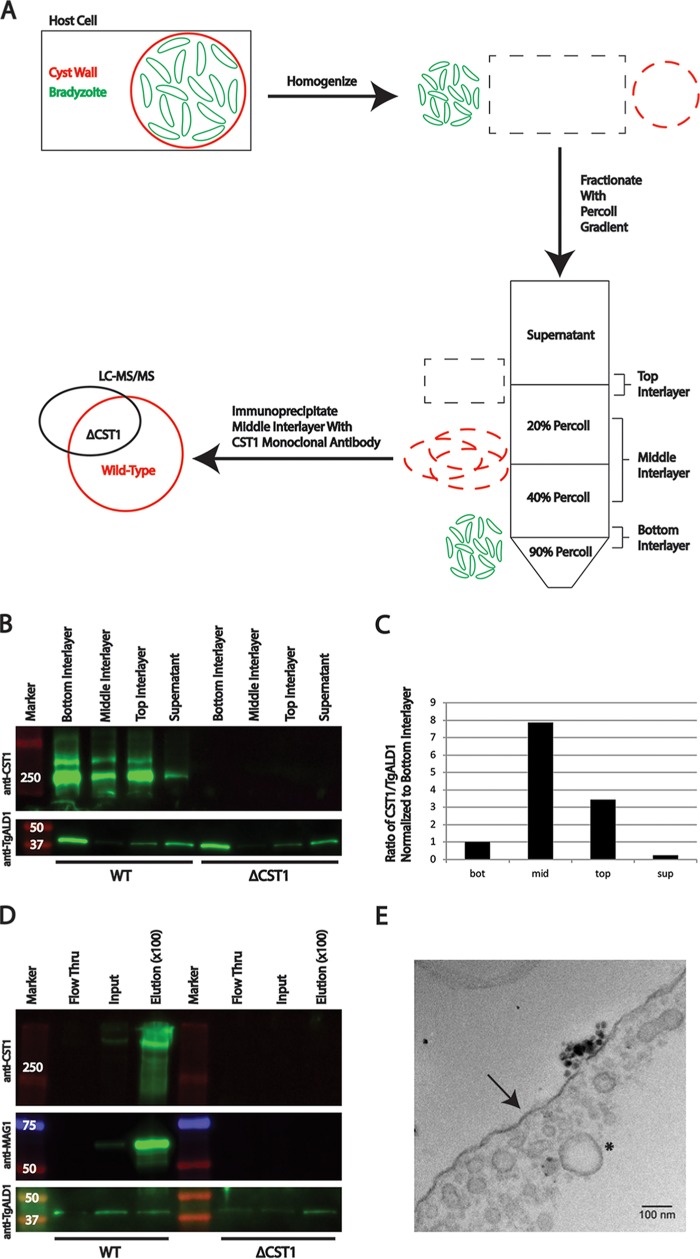
Enrichment of the cyst wall fraction using Percoll gradient and immunoprecipitation with anti-CST1 antibody. (A) Schematic showing the enrichment of cyst wall fragments from the parasite body using Percoll stepwise gradient centrifugation and anti-CST1 immunoprecipitation. *In vitro*-induced bradyzoite samples were lysed and fractionated on a 90-40-20% Percoll gradient. The middle interlayer between the 40% and 20% Percoll solutions was used for immunoprecipitation with anti-CST1 MAb-coated magnetic beads. Proteins in the pulldowned fragments were separated by SDS-PAGE followed by LC-MS/MS analysis. CST1-KO parasites served as a negative control to subtract the nonspecific binding of proteins to beads in the absence of CST1. (B) Immunoblot of equivalent volumes from Percoll gradient interlayers from Pru*Δku80Δhxgprt* and CST1-KO bradyzoite samples using anti-CST1 (1:500) antibody to detect the cyst wall fragment or anti-ALD1 (1:500) to detect the parasite body. Donkey anti-rabbit 680 (Li-Cor) and Donkey anti-mouse 800 (Li-Cor) secondary antibodies were used at a 1:1,000 dilution. The labeled membranes were captured with the Odyssey Fc system (Li-Cor). The Bottom Interlayer is the interlayer between the 90% and 40% Percoll solution, the Middle Interlayer is the interlayer between the 40% and 20% Percoll solution, the Top Interlayer is the interlayer between 20% Percoll and homogenate, and the Supernatant is the fraction above the 20% Percoll solution layer as depicted in Fig. 1A. Molecular weight markers (in kilodaltons) are shown in the leftmost lane. (C) Relative enrichment score of the CST1/ALD1 ratio was calculated using the signal intensities in Fig. 1B, determined with Li-Cor Image Studio Lite software. All values were normalized to the CST1/ALD1 ratio of the bottom interlayer. (D) Immunoblot of the immunoprecipitation performed on the middle interlayer (mid). Proteins from the middle interlayer were probed with anti-CST1 (1:500), anti-MAG1 (1:500), and anti-ALD1 (1:500) antibody. Flow Thru is the flowthrough fraction that did not bind to CST1-coated beads after overnight incubation. Input samples for immunoprecipitation (Input) and proteins eluted from the beads (Elution) are shown. Because the elution was performed with a relatively small volume, the eluted samples were enriched ∼100 times compared to the Input and Flow Thru samples. Note the enrichment for the highly glycosylated form of CST1 (>250 kDa) with the salmonE antibody. (E) Magnetic beads used for immunoprecipitation of the WT parasites were fixed, embedded in resin, stained, and sectioned for electron microscopy. The black arrow points to a structure that resembles an intact cyst membrane, while the black asterisk highlights vesicles reminiscent of cyst wall architecture.

To test whether Percoll gradients could separate bradyzoites from cyst wall fragments, HFF cells containing cysts were ruptured using a 27-gauge needle, followed by passage through a 6-μm-clearance ball-bearing homogenizer. This material was then loaded onto a 90-40-20% Percoll gradient for centrifugation (Fig. 1A). The cyst wall protein CST1 (7) was detected in each fraction obtained from the Percoll gradient, whereas the T. gondii cytosolic protein aldolase 1 (ALD1) was detected predominantly in the bottom interlayer (Fig. 1B). By comparing the ratio of the signal intensity from CST1 versus ALD1 in each fraction, the middle interlayer was found to contain the highest ratio of cyst wall fragment proteins compared to parasite body/cytosolic protein contamination (Fig. 1C). Therefore, the middle fraction was selected for further purification with anti-CST1 immunoprecipitation using magnetic beads. After magnetic separation and elution of proteins from the anti-CST1-coated beads, the fraction bound to the beads was enriched for CST1 and MAG1 (matrix antigen 1), but not for ALD1 (Fig. 1D). Anti-MAG1 monoclonal antibody was used as an additional marker for cyst wall fragments. As previously characterized, CST1-KO parasites have a defect in bradyzoite differentiation (7); thus, MAG1 expression was lower in these parasites than in the wild-type (WT) parasites (Fig. 1D, input lane). Since cytosolic protein contamination levels between the WT and CST1 knockout parasites (ΔCST1) after bead elution were similar (Fig. 1D, elution lanes), the sample from CST1-KO parasites was used as a negative control in subsequent mass spectrometry analyses, as this sample contains parasite proteins bound to the coated beads nonspecifically (see the strategy in Fig. 1A). Finally, the fractions bound to the beads were further analyzed by transmission electron microscopy (TEM) to confirm immunoprecipitation of cyst wall fragments. The purified fragments from WT bradyzoite cysts contained membranous structures with small vacuoles (approximate diameter, ∼20 to 100 nm) (Fig. 1E), which bear similarities to the cyst wall structure observed in TEM analysis of intact tissue cysts (4, 7); however, this purified membranous material lacked the electron-dense material and tubules that have been observed in intact cysts.

### Protein identification of cyst wall fragments from *in vitro*-induced bradyzoite cysts by LC-MS/MS.

Proteins from the cyst wall enriched fraction were identified using liquid chromatography-tandem mass spectrometry (LC-MS/MS). Top hits detected from the WT sample, but not from the CST1-KO sample, contained several known cyst wall proteins, including CST1, BPK1, MAG1, MCP4, GRA2, GRA3, and GRA5 (Table 1), which validate this proteomic approach for identifying cyst wall components. A total of 38 proteins were above the threshold of at least 10-fold-higher spectral counts in the WT sample compared to the CST1-KO sample. These top hits shared patterns of high expression in the bradyzoite stage (M4 *in vivo* bradyzoite expression profile [6], retrieved from ToxoDB, percentile >75%, Table 1). Using the SignalP 4.1 webserver and gene models from ToxoDB (version 40), 21 of the 38 proteins that made the cutoff are predicted to contain signal peptides (see Data Set S1 in the supplemental material). Similarly, NetNGlyc 1.0 and NetOGlyc 4.0 webserver analyses predict that a subset of these proteins are glycoproteins (Data Set S1). A replicate cyst wall sample was also analyzed, and the same proteins were identified in this sample.

**TABLE 1:** Top hits obtained from LC-MS/MS analysis of cyst wall fractions^*a*^

ToxoDB ID (TgME49_)	Protein	WT/CST1-KO	Bradyzoite percentile	Other studies (PMID)
264660	CST1	4,885	98.2	24385904
253330	BPK1	134	99.5	23291621
203290	GRA34	111	98.8	27486190
251540	GRA9	82	97.1	15491588
270240	MAG1	74	99.3	7808478
209755	Hypothetical protein	73	99.0	NA
208740	MCP3	63	98.5	19901027
204340	Hypothetical protein	55	90.0	19218426
213067	GRA36	52	92.3	27486190
258870	Hypothetical protein	45	93.0	NA
227280	GRA3	44	97.9	8195173
221620	Beta-tubulin, putative	38	91.0	19886702
239740	GRA14	34	94.5	18765740
208730	MCP4	31	96.3	22021236
319340	CST5/GRA49 (this study)	27	97.3	21920448
254720	GRA8	26	93.7	10613696
200360	Hypothetical protein	25	98.8	23027733
286450	GRA5	23	99.7	8515776
203600	CST2/GRA47 (this study)	22	81.4	30362762
222170	GRA17	21	93.9	25974303
203310	GRA7	21	98.6	9566518
254470	MYR1	20	82.2	26838724
260520	CST6/GRA50 (this study)	20	96.2	NA
220240	GRA31	19	84.2	27486190
226380	GRA35	18	95.5	27486190
297880	GRA23	17	92.6	23583316
219820	Polyubiquitin UbC, putative	17	96.4	NA
270320	PPM3C	16	92.3	28556455
227620	GRA2	15	99.3	8384696
263300	Porin protein, putative	15	88.2	NA
230705	CST3/GRA48 (this study)	15	83.5	NA
288650	GRA12	13	99.0	18840447
247440	GRA33	13	94.4	27486190
290700	GRA25	13	93.2	24711568
279100	MAF1-related protein	12	84.5	26920761
310780	GRA4	12	95.9	1362450
269690	GRA29	11	96.1	27486190
261650	CST4 (this study)	11	92.1	NA

a The proteins identified are ranked in order of relative abundance using total spectrum counts in the WT strain and comparing to the total spectrum counts in the CST1-KO strain (WT + 1/CST1-KO + 1). An arbitrary WT/CST1-KO cutoff value of ≥10 was used to report top hits from cyst wall fractions in this table. All proteins identified from LC-MS/MS are provided in Data Set S1 in the supplemental material. Bradyzoite percentile values were obtained from transcriptomic data deposited into ToxoDB, Version 40 (Bradyzoite *in vivo* transcriptome [M4] [6]). Other studies in which proteins on this list were previously described are referenced by PubMed ID number (PMID). NA, not available.

10.1128/mBio.00469-19.7 DATA SET S1 LC-MS/MS results obtained from wild-type and CST1-KO cyst wall preparations, indicating the amount of peptides identified from *Toxoplasma* and human proteins. *Toxoplasma* proteins were scored by peptide enrichment in the wild-type sample compared to the CST1-KO sample, and webserver analyses were also performed to identify predicted signal peptides (SignalP 4.1), N-linked glycosylation (NetNGlyc 1.0), and O-linked glycosylation (NetOGlyc 4.0). Download Data Set S1, XLSX file, 0.1 MB. Copyright © 2019 Tu et al. 2019 Tu et al. This content is distributed under the terms of the Creative Commons Attribution 4.0 International license.

Of the 38 identified proteins that made the cutoff, 16 proteins are currently annotated as hypothetical proteins in ToxoDB (version 40), with 8 of these hypothetical proteins being novel dense granule proteins (GRAs) identified in recent proteomic studies of the parasitophorous vacuole utilizing proximity-based biotinylation approaches (12, 13). Interestingly, the majority of the proteins identified in the cyst wall fraction are GRA proteins that have been shown, in the tachyzoite stage, to localize to the PV/PVM space in the GRA1, GRA13, GRA17, and GRA25 interactomes (12, 13). On the other hand, seven of the proteins identified in cyst wall fractions were not identified in the aforementioned studies on tachyzoite parasitophorous vacuoles.

### Novel cyst wall fraction proteins localize to the cyst wall, cyst matrix, and dense granules.

**FIG 2:**
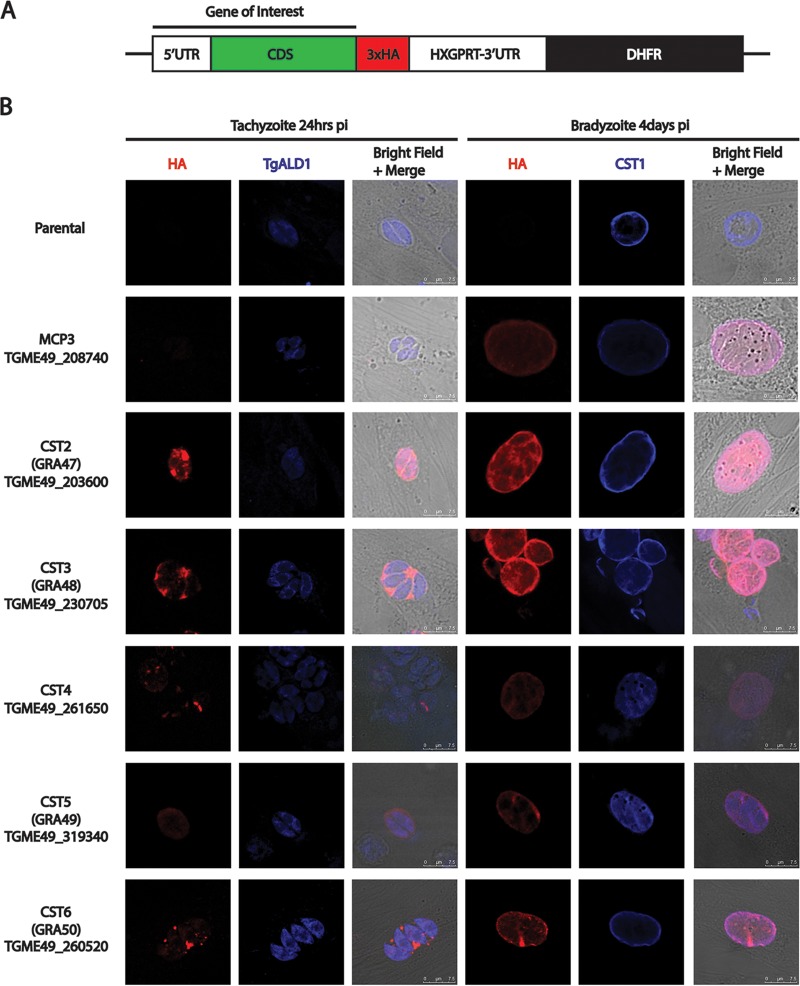
Identified proteins localize to the cyst wall and matrix of *in vitro*-induced bradyzoites. (A) Schematic diagram of the constructs used for tagging the C termini of the genes of interest with 3xHA. (B) IFA of 3xHA-tagged proteins and ALD1 (parasite cytosol marker) or CST1 (cyst wall marker) were performed in tachyzoite- or bradyzoite-infected samples *in vitro*, respectively. For tachyzoite staining, parasites were cultured for 24 h after infection in normal culture conditions, fixed, and stained. For bradyzoite staining, parasites were induced with pH 8.2 medium with CO_2_ depletion after a 2-h infection period with bradyzoite induction lasting 4 days. For both tachyzoites and bradyzoites, 3xHA is shown in red. For tachyzoites, ALD1 is shown in blue; for bradyzoites, CST1 is shown in blue.

To validate whether the uncharacterized hypothetical proteins from the cyst wall pulldown were novel cyst wall proteins, genes that were not detected from GRAomic data sets (12, 13) (TgME49_230705, TgME49_261650) or had high expression within the M4 *in vivo* bradyzoite transcriptome data set deposited in ToxoDB (6) (MCP3, TgME49_260520, TgME49_203600, TgME49_319340) were chosen to be expressed in the parasite with a 3xHA epitope tag driven by their native promoters (Fig. 2A).

MCP3 protein sequence analysis revealed several microneme adhesive repeat domains on this protein that do not have the potential to bind sialic acid due to the lack of conserved threonines in these domains (14). Similar to MCP4 (6), MCP3 expression was low in tachyzoites but high in bradyzoites, showing localization to the bradyzoite cyst wall and colocalization with CST1 (Fig. 2B). TgME49_203600, TgME49_230705, TgME49_319340, and TgME49_260520 all show localization in the tachyzoite matrix as well as the cyst matrix and cyst wall. TgME49_261650, which contains WD-40 repeat-like domains, was shown to localize to a subcellular compartment in tachyzoites as well as to the cyst matrix and the cyst wall in the bradyzoite stage (Fig. 2B). Of these six genes, all but TgME49_261650 localized to the dense granules, colocalizing with GRA1 in extracellular tachyzoites (see Fig. S1 in the supplemental material). As these hypothetical proteins demonstrated localization to the cyst wall under immunofluorescence, we renamed them with “CST” names, and numbered them in the order in which they were characterized (Table 1). Based on the colocalization of these proteins with dense granule protein GRA1, many of these new CST proteins represent a subset of GRA proteins that localize to the cyst wall. New CST proteins that localize to the dense granule have also been given GRA protein designations (with the starting number for these new GRA proteins being based on the last number used for GRA proteins described in the literature (15) (Table 1). Localization of these proteins was not observed to change *in vitro* with prolonged (more than 4 days) bradyzoite induction (data not shown). These validation data on the hypothetical proteins identified by the Percoll fractionation followed by CST1 immunoprecipitation confirmed that this technique was able to enrich cyst wall fragments for proteomic analysis.

10.1128/mBio.00469-19.1 FIG S1 Colocalization of GRA1 and HA-tagged hypothetical proteins. IFA of HA-tagged proteins (red) and GRA1 (green) in extracellular tachyzoites (nuclei labeled with DAPI [blue]). Colocalization was observed between GRA1 and each HA-tagged protein, with the exception of CST4. Download FIG S1, PDF file, 0.8 MB. Copyright © 2019 Tu et al. 2019 Tu et al. This content is distributed under the terms of the Creative Commons Attribution 4.0 International license.

### CST2 is important for the establishment of chronic infection *in vivo*.

**FIG 3:**
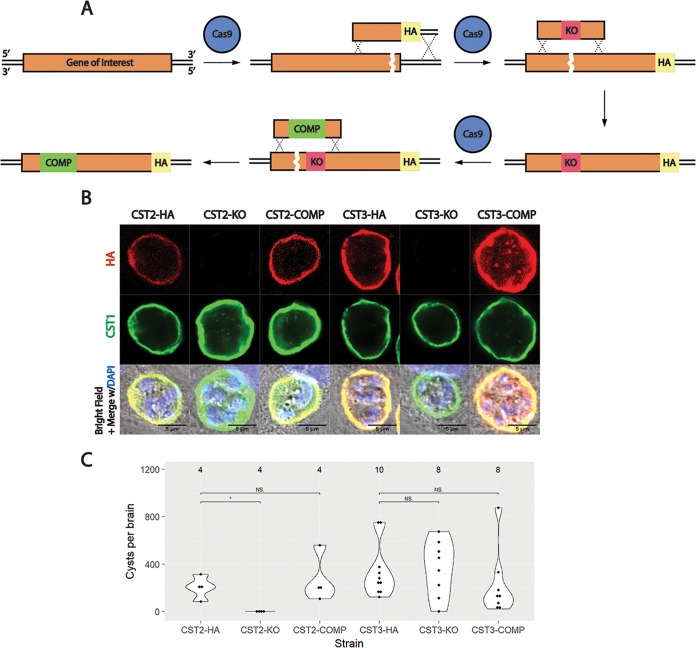
Characterization of novel cyst wall proteins CST2 and CST3. (A) Schematic diagram of the tagging, deletion, and complementation of the CST2 and CST3 loci using CRISPR/Cas9. The C termini of the genes of interest were endogenously tagged with 1xHA to generate CST2-HA and CST3-HA. Subsequently, premature stop codons were inserted into the N termini of the CST2-HA and CST3-HA loci. Expression of CST2-HA or CST3-HA was restored by complementing N-terminal sequences with synonymous mutations into CST2-KO or CST3-KO. (B) IFA of CST2 and CST3 after tagging the genes with 1xHA, deletion, and complementation. 1xHA is shown in red, and CST1 is shown in green. (C) Number of cysts recovered from the brains of C57BL/6 mice after infecting with HA, KO, or COMP strains of CST2 or CST3. Values above each group indicate the number of mice sacrificed. N.S., not significant; *, *P* < 0.05.

To further characterize the role of two of these hypothetical proteins in cyst wall formation, CST2 (TgME49_203600) and CST3 (TgME49_230705) genes were knocked out in T. gondii using a recyclable selection strategy (16). First, these genes were tagged endogenously at their C termini with a 1xHA epitope tag (17). Then, donor sequences containing the N terminus of CST2 and CST3 flanking two tandem stop codons (Table S1) were cotransfected with a guide RNA targeting the N terminus of CST2 or CST3 into CST2-HA or CST3-HA parasites, respectively, to generate the knockout (KO) strains (Fig. 3A). To complement the knocked-out genes, a guide RNA targeting a different site on the N terminus of each gene was cotransfected with donor sequences containing synonymous mutations in the N terminus of CST2 or CST3 to allow for verification of the integrated complement sequences by Sanger sequencing (Fig. S2A). Immunofluorescence with anti-HA antibody confirmed the tagging, deletion, and complementation of each gene (Fig. 3B).

10.1128/mBio.00469-19.2 FIG S2 Sequencing analysis and plaque assays from CST2 and CST3 strains. (A) Sanger sequencing chromatograms of the genomic DNA of each KO and COMP strain. Note the incorporation of a tandem stop codon into the KO strain, and the restoration of the original coding sequence using synonymous mutations. (B) Representative images from plaque assays of each strain are shown on the left, and quantification of plaque size from each strain is shown on the right. The number of plaques analyzed per strain is provided above each violin plot. Download FIG S2, PDF file, 1.2 MB. Copyright © 2019 Tu et al. 2019 Tu et al. This content is distributed under the terms of the Creative Commons Attribution 4.0 International license.

10.1128/mBio.00469-19.5 TABLE S1 Oligomers used as donor DNA. Download Table S1, DOCX file, 0.01 MB. Copyright © 2019 Tu et al. 2019 Tu et al. This content is distributed under the terms of the Creative Commons Attribution 4.0 International license.

Following genetic manipulation, the overall fitness of the knockout parasites were analyzed using plaque assays. CST2-KO and CST3-KO parasites did not create significantly different plaque sizes compared to their parental or complement lines (Fig. S2B), suggesting that CST2 and CST3 do not play a role in parasite invasion, replication, or egress. The localizations of several known cyst wall proteins were also probed within these knockout strains. No difference in the immunofluorescence localization of CST1, MAG1, and MCP4 was seen between Pru*Δku80Δhxgprt* and CST2-KO or CST3-KO parasites *in vitro* (Fig. S3).

10.1128/mBio.00469-19.3 FIG S3 *In vitro* cyst wall protein localization in CST2-KO and CST3-KO parasites. Immunofluorescence assay images of CST1, MAG1 (A), and MCP4 (B) localization in the Pru, CST2-KO, and CST3-KO strains *in vitro*. Primary and secondary antibody dilutions of 1:500 were used for each label. Download FIG S3, PDF file, 1.0 MB. Copyright © 2019 Tu et al. 2019 Tu et al. This content is distributed under the terms of the Creative Commons Attribution 4.0 International license.

To measure cyst burdens, C57BL/6 mice were infected intraperitoneally with 1,000 parasites of the CST2 or CST3 parasite strains. Mouse survival rates after 30 days at this dosage were not significantly different between the KO strains and their parental and complement strains (Fig. S4A). However, the numbers of cysts recovered from CST2-KO parasites were undetectable compared to CST2-HA and CST2-COMP parasites (Fig. 3C). On the other hand, CST3-KO parasites did not show any significant differences in cyst numbers or cyst sizes between its parental and complement strain (Fig. 3C; Fig. S4B).

10.1128/mBio.00469-19.4 FIG S4 Mouse survival and cyst sizes from CST2 or CST3 T. gondii infection. (A) Survival curves from mouse virulence assays of mice infected with 1,000 parasites of either CST2 or CST3 strains. *n* = 10 for each strain used. No significant differences were observed between any of the groups of mice infected. No mortality was observed for PBS control. (B) Measurement of cyst size of CST3 strains obtained from mouse brains. The numbers of cysts obtained per strain is provided above each violin plot. No significant differences in size were observed between the strains. (C) Survival curves from mouse virulence assays of mice infected with 100,000 parasites from either Pru, CST2-HA, CST2-KO, or CST2-COMP T. gondii. *n* = 10 for each strain used. No mortality was observed in mice infected with the CST2-KO strain. The reported *P* value was generated by comparing CST2-KO to all the other strains. Download FIG S4, PDF file, 0.6 MB. Copyright © 2019 Tu et al. 2019 Tu et al. This content is distributed under the terms of the Creative Commons Attribution 4.0 International license.

**FIG 4:**
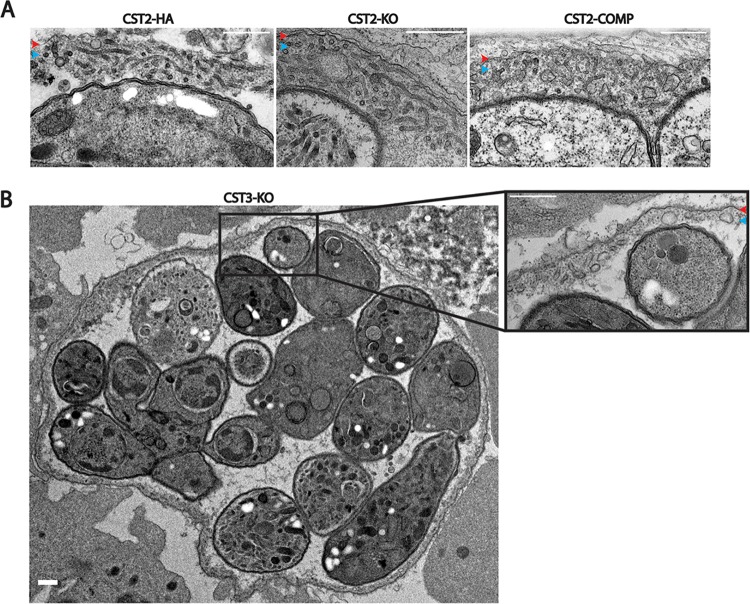
Ultrastructure of cysts generated from CST2-KO and CST3-KO parasites. (A) Transmission electron micrograph of *in vitro* cysts containing CST2-HA, KO, and COMP parasites. (B) Transmission electron micrograph of an *in vivo* cyst containing CST3-KO parasites. Red arrowheads point to cyst membranes, while blue arrowheads point to cyst walls. Bars, 500 nm.

Given that no cysts were detected after the mice were infected with CST2-KO parasites, we hypothesized that CST2 might be involved in the formation of a functional cyst wall. The ultrastructure of *in vitro* CST2-HA, KO, and COMP cysts were examined by electron microscopy to further assess the role of CST2 in cyst morphology. Strikingly, no differences in the *in vitro* cyst wall structure between the CST2-HA, KO, and COMP strains could be detected (Fig. 4A). Similarly, CST3-KO *in vivo* cysts displayed normal cyst wall ultrastructure with an amorphous granular layer underneath the cyst membrane (Fig. 4B). To further investigate why CST2-KO cysts were not detected in the mouse brain during chronic infection, the virulence of the CST2 strains was assessed by infecting C57BL/6 mice using a high intraperitoneal inoculum (100,000 parasites) of the Pru, CST2-HA, CST2-KO, or CST2-COMP T. gondii strains. Mice were monitored for 30 days, and 60% to 70% of the mice infected with parasites containing an intact CST2 locus succumbed to acute infection, but all mice infected with CST2-KO survived (Fig. S4C). These data suggest that attenuation of CST2-KO parasite virulence during acute infection could contribute to the lack of CST2-KO cysts observed in the brains of mice with chronic infection.

## DISCUSSION

The cyst wall is present in cysts and is absent in the tachyzoite parasitophorous vacuole. However, after bradyzoite differentiation, little is known about how the cyst wall is built, the composition of the cyst wall, how the cyst membrane is built or derived from modifications to the PVM, or how the cyst membrane differs from the tachyzoite stage PVM. Here, we provide the first proteomic analysis of the composition of the cyst wall. The cyst wall fraction we purified and analyzed contained previously described cyst wall proteins (e.g., CST1 and MAG1) that localized to the cyst wall and/or cyst matrix as well as uncharacterized hypothetical proteins. We characterized the localization of these hypothetical proteins by expressing the gene of interest with a 3xHA epitope tag, which revealed that these proteins are novel cyst wall and cyst matrix localizing proteins. All of the identified genes above our arbitrary cutoff have a bradyzoite mRNA expression profile over the 75th percentile (M4 *in vivo* RNAseq, ME49 *in vitro* RNAseq) in ToxoDB. We confirmed that these transcripts are also expressed at the protein level in our epitope-tagged transgenic parasites during the bradyzoite stage. To expand on the functions of these proteins at the cyst wall, we knocked out two of these proteins, CST2 and CST3, using a recyclable selectable marker strategy (16) and characterized these transgenic parasites for their ability to form cysts. While the deletion strains do not display an abnormal phenotype with respect to growth or cyst formation *in vitro*, CST2-KO parasites are markedly less virulent during acute infection in mice (see Fig. S4C in the supplemental material). This may reflect an inability to establish chronic infection *in vivo*, and it is possible that the reduced cyst burden observed in infection with CST2-KO parasites may be partially due to reduced dissemination of the parasite to the central nervous system during acute infection. These data demonstrate that while the parasite fractions purified in this study are enriched in the cyst membrane and cyst wall, the functions of these proteins may be relevant to both the tachyzoite and bradyzoite life stages.

GRA proteins that have been previously characterized in the tachyzoite stage are also detected in the *in vitro* bradyzoite cyst wall fraction. Transcriptomic data of *in vivo* cysts indicate that these GRA genes are also actively transcribed in *in vivo* bradyzoites (M4 *in vivo* bradyzoite transcriptome data set [6] from ToxoDB). Recent studies that extended the dense granule proteome through the use of BirA* (12, 13) and APEX tags (13) identified 28 out of 38 proteins that were found in our cyst wall fraction. These proteins include MAG1, CST2, CST5, CST6, hypothetical proteins (TgME49_258870, TgME49_204340, and TgME49_200360), dense granule proteins (GRA2, GRA3, GRA4, GRA5, GRA7, GRA8, GRA9, GRA12, GRA14, GRA17, GRA23, GRA25, GRA29, GRA31, GRA33, GRA34, GRA35, and GRA36) (12, 18), a rhoptry PP2C, a MAF1-related protein (19), and MYR1 (20). Notably, TgME49_200360 was first identified in a study of the T. gondii basal complex, where it was shown to interact with MORN1 and localize to dense granules and the PV lumen (21). To fully describe the hypothetical proteins characterized in this study, we have assigned dual names to them to designate their localization to the cyst wall in the bradyzoite stage (CST) and the dense granule secretory pathway (GRA) from where they originate (Table 1). The use of “CST” defines a subset of proteins that are involved in the cyst wall and is consistent with current naming conventions (22, 23). Using this nomenclature, previously characterized dense granule proteins such as GRA5 may also be considered “CST” proteins given their confirmed localization to the cyst wall; however, as per convention, the names of these and other gene products already identified and established in the literature are not being assigned a secondary name. Previous characterizations of GRA proteins have mainly focused on tachyzoites, and our data suggest that the functions of these proteins in the bradyzoite stage should also be investigated to fully characterize the roles of these proteins in the biology of T. gondii.

GRAs have been characterized to play a role in small molecule trafficking through the PVM in the tachyzoite stage (24). The presence of the GRA23 and GRA17 in the cyst wall proteome suggests that small molecule transport is also active in cysts, even with the presence of the cyst wall. Previous reports show that the cyst membrane and cyst wall are permeable to small molecules less than 10 kDa (25), which supports the idea that there is active transport of small molecules through the cyst membrane and cyst wall. In addition to small molecule trafficking, GRA proteins are known to play roles in forming the intravacuolar network (IVN) in the tachyzoite PV. Ultrastructural analysis suggests that some proteins are secreted from the posterior end of bradyzoites through a vesicular network in the cyst matrix, termed the intracystic network (ICN) (25). Whether the ICN is a new structure in cysts or whether the ICN is a modified IVN is not known. Our data show that the cyst wall fraction contained several GRA proteins (GRA2, GRA4, GRA9, and GRA12) that exclusively localize to the IVN membranes in the tachyzoite PV. These findings support the idea proposed by Lemgruber et al. (25) that the tachyzoite parasitophorous vacuole IVN structures and the cyst ICN structures contribute to cyst wall development.

The translocon component MYR1 (20), which is a protein required for the translocation of GRA16 (26) and GRA24 (27) from the tachyzoite PV into the host cell nucleus, was also identified in the cyst wall enriched fraction. A recent publication elegantly demonstrated that GRA16 and GRA24 do not seem to translocate beyond the cyst wall when expressed ectopically 5 days after bradyzoite conversion *in vitro* (28). Work in our laboratory agrees with this observation, with the caveat that upon infection with *in vitro-*derived bradyzoites, GRA16 is detected in the host cell nucleus at 2 days postinfection, but not at later time points (unpublished data), suggesting that effector export may be an event that occurs only during the early stages of bradyzoite invasion and cyst development. The serine protease ASP5, which plays a role in the translocation of GRA16 and GRA24 (29, 30), is known to operate in the bradyzoite stage to form proper cyst walls (31), further suggesting that translocation machinery is readily available even in the bradyzoite stage. Future work regarding the extent and consequences of effector export within *in vitro* and *in vivo* cysts will aid in understanding the host-parasite interaction of the latent bradyzoite stage.

TgME49_279100, a MAF1-related protein classified as a MAF1a paralog, was detected in the enriched cyst wall fraction. MAF1b paralogs, but not MAF1a paralogs, have been shown to be responsible for the HMA phenotype (19, 32). Our data are in agreement with the lack of HMA observed in type II strains, as we did not detect the MAF1b paralog in the cyst wall fraction (19, 32).

Analysis of the cyst wall preparation also found TGME49_263300 (a putative homolog of TOM40), TGME49_219820 (a putative homolog of polyubiquitin C), and TGME49_221620 (a cytoskeletal protein tubulin). We consider these hits as likely nonspecific binding proteins that result from low stringent washing conditions in our enrichment process, which lacks detergent so as to maintain the membranous structure of the cyst wall. However, it should be noted that parasite tubulin has been also identified in other proteomic approaches, such as the proximity-based biotinylation of tachyzoite parasitophorous vacuolar proteins by GRA proteins (12), suggesting that they may have novel roles in the parasitophorous vacuolar space.

Overall, the cyst wall purification method presented here successfully identified proteins that are secreted into the bradyzoite cyst matrix. We characterized six hypothetical proteins as novel cyst matrix and cyst wall proteins, demonstrating that our current strategy using *in vitro*-induced cysts is powerful in identifying cyst wall proteins. While it is not completely possible to exclude proteins identified in the cyst wall enriched fraction as having originated from proteins expressed in the tachyzoite stage during the course of differentiation, the mRNA expression data of these genes strongly suggest that these proteins are expressed in the bradyzoite stage. As we utilized *in vitro-*derived bradyzoite samples, with the rationale of generating sufficient amounts of cysts and starting material for fractionation, our data set did not include the cyst wall protein BCP1 (TgME49_203450), which has been shown to exclusively localize to the cyst wall of *in vivo*-derived tissue cysts (33). Future work to identify additional cyst wall components that exclusively localize to the cyst wall of *in vivo-*derived cysts will likely require a breakthrough in the amounts of tissue cysts that can be harvested from the average chronically infected mouse or rat brain. Once sufficient amounts of tissue cyst samples can be isolated *in vivo*, our fragmentation and cyst wall purification steps could be useful in removing host and parasite cytosol fractions to perform targeted cyst wall proteomic analyses.

As the CST1 pulldown in our approach was performed with membrane fragments and not soluble proteins, the data set we obtained revealed not only CST1-interacting proteins but also proteins that indirectly associate with CST1 through intact cyst wall structures. This enabled us to identify cyst wall and cyst matrix proteins in cysts. However, for the identification of interactions among the identified proteins and to understand how the whole cyst wall structure is constructed, further analysis that can detect protein proximity is required. Proximity-based biotinylation approaches, such as the use of the BirA* tag, has been previously shown to be a feasible approach in *Toxoplasma* (12, 34). A BirA*-based cyst wall interactome is currently being studied, and we expect proteins on our list to be validated using the BirA* approach (unpublished data). Combining data sets obtained from both this report and proximity-based interactome approaches will clearly aid understanding of cyst wall biology, the mechanisms for building this structure, and key characteristics of latent infection of T. gondii.

## MATERIALS AND METHODS

### Parasite culture.

Throughout this study, Pru*Δku80Δhxgprt* wild-type parasites and Pru*Δku80Δcst1* (7) (CST1: TgME49_264660) were cultured with human foreskin fibroblast (HFF) (ATCC:CRL-1634; Hs27) host cells, as described elsewhere (35). Bradyzoite induction was performed as described elsewhere (35). Briefly, parasites at a multiplicity of infection (MOI) of 0.5 to 1.0 were inoculated into a confluent HFF monolayer. After a 2-h invasion window at 37°C in a CO_2_ incubator, the medium was changed to DMEM with 50 mM HEPES (pH 8.2) without NaHCO_3_, supplemented with 1% FBS, penicillin and streptomycin, and incubated in a humid 37°C incubator without CO_2_. Induction medium was changed every 2 days.

### Cyst wall fraction enrichment.

To generate *in vitro* cysts, five confluent HFF monolayers in 150-mm dishes were infected with freshly ruptured Pru*Δku80Δhxgprt* or Pru*Δku80Δcst1* (negative-control) tachyzoites. After 8 days of bradyzoite induction, infected host cells were rinsed with ice-cold PBS containing phosphatase inhibitors (5 mM NaF and 2 mM Na_3_VO_4_) and a protease inhibitor cocktail (Roche) (homogenate buffer), scraped in 75 ml homogenate buffer, and ruptured by passage through a 27-gauge needle five times. To further break the *in vitro*-induced cysts, the homogenate was passed through a 6-μm-clearance ball-bearing homogenizer (Isobiotech, Heidelberg, Germany) 10 times.

To separate the parasite body from cyst wall fragments, solutions of 90% Percoll (first Percoll layer), 40% Percoll (second Percoll layer), and 20% Percoll (third Percoll layer) was created using 10× PBS diluted to 1×. Then, a stepwise Percoll gradient solution was layered using 1 ml of each Percoll solution into 15-ml tubes, and 6 ml of the homogenized cell fragments was placed on top. The samples were centrifuged at 1,250 × *g* for 15 min in 4°C. Three interlayers (bottom, middle, and top) and the supernatant were collected for the further analysis. The middle interlayer (interlayer between the second and third Percoll layers) was harvested for cyst wall enrichment by anti-CST1 immunoprecipitation.

### Immunoseparation of the CST1-containing fragments.

Protein L-coated magnetic beads (Pierce) were cross-linked with 100 μg salmonE (anti-CST1) monoclonal IgE antibody with BS^3^ cross-linking reagent according to the manufacturer’s instructions. Coated beads were divided in half and used for the immunoprecipitation of WT and *Δcst1* parasites separately.

A total of 10 ml from the middle fraction was added to binding buffer (PBS containing 2% bovine serum with phosphatase and protease inhibitors) and salmonE anti-CST1 MAb-coated magnetic beads. The beads were incubated overnight at 4°C with gentle rotation. The beads were magnetically separated and rinsed once with 1 ml binding buffer, five times with 1 ml homogenate buffer, and once with 1 ml PBS. The proteins in the immunoprecipitated cyst wall fractions were eluted with Laemmli buffer and separated by SDS-PAGE. Whole lanes were analyzed by mass spectrometry.

### Mass spectrometry analysis.

Proteomic analysis of the protein sample separated by SDS-PAGE was performed as described elsewhere (36). One lane of the sample was split into 24 pieces, and the gels were diced into 1-mm cubes. The proteins were processed for in-gel digestions, and peptide solutions were separated with liquid chromatography, ionized by electrospray ionization (LC-ESI). Identification of peptide mass and charge was performed by LTQ-Orbitrap-MS/MS, and peptide sequences were identified using Scaffold4 software. For a database search to match the detected spectra to identified proteins, a T. gondii protein database was built from the type II ME49 strain genome on ToxoDB version 12.

To identify cyst wall enriched proteins with high confidence, we compared the proteins obtained from Pru*Δku80Δhxgprt* parasites and Pru*Δku80Δcst1* parasites. The total assigned spectrum counts for each protein were normalized to the total detected spectrum counts, and enrichments were ranked by an enrichment score (WT spectrum counts + 1/KO spectrum counts + 1). Full spectrum counts for the proteins are available in Data Set S1 in the supplemental material. The proteomic data have been deposited in ToxoDB.org.

### Second copy expression of tagged hypothetical proteins.

To express C-terminal 3×HA-tagged proteins for the uncharacterized genes identified, the genomic locus of the gene of interest, from the promoter region (1.5 kb to 2 kb upstream of the start codon) to the coding sequence (excluding the stop codon) was cloned into a pLIC-3HA-DHFR plasmid backbone as described elsewhere (35). A full list of primers used is available in Table S2. Pru*Δku80Δhxgprt* tachyzoites were transfected with 5 μg plasmid DNA with electroporation in incomplete cytomix. Transfected parasites were selected for with 1 μM pyrimethamine and subcloned by limiting dilution.

10.1128/mBio.00469-19.6 TABLE S2 Primers used for cloning or sequencing. Download Table S2, DOCX file, 0.01 MB. Copyright © 2019 Tu et al. 2019 Tu et al. This content is distributed under the terms of the Creative Commons Attribution 4.0 International license.

### Endogenous gene tagging, deletion, and complementation.

To epitope tag CST2 (TgME49_203600) and CST3 (TgME49_230705), p-HXGPRT-Cas9-GFP containing single guide RNAs (sgRNAs) targeting the C termini of the genes of interest were prepared as previously described (16). Donor DNA consisting of 100-bp oligonucleotides (ThermoFisher) of the forward and reverse sequences of the 1xHA epitope flanked by the gene’s C terminus and 3′UTR were cotransfected with 20 μg of circular p-HXGPRT-Cas9-GFP at a 100:1 mol ratio into Pru*Δku80Δhxgprt*. After transfection, transgenic parasites were selected with 25 μg/ml mycophenolic acid and 50 μg/ml xanthine for 10 days before subcloning immediately.

To generate knockout parasites, a sgRNA with a target sequence near the gene’s start codon was cloned into p-DHFR-Cas9-GFP. This Cas9 construct was cotransfected with 100-bp donor oligonucleotides containing two tandem stop codons flanked by the gene’s 5′UTR and N terminus into CST2 or CST3-HA parasites. Transfected parasites were selected with 1 μM pyrimethamine for 10 days before subcloning.

Complement parasites were generated using the same strategy described above, choosing a separate sgRNA cloned into p-HXGPRT-Cas9-GFP with a target site near the tandem stop codons. All primer and oligonucleotide donor sequences used to generate these strains are provided in Tables S1 and S2.

### Immunofluorescence of uncharacterized proteins.

To characterize the localization of the epitope-tagged proteins and their bradyzoite expression, HFF monolayers were infected and cultured for 24 h before fixing (tachyzoites) or induced to become bradyzoites for 4 days before fixing. Fixed cells were stained for immunofluorescence assays (IFAs) as described elsewhere (35). HA-tagged proteins were detected by anti-HA rat monoclonal antibody 3F10 (Sigma; 1:250), parasite cytosol by anti-ALD1 rabbit antibody (1:500) (a kind gift from Kentaro Kato, University of Tokyo) (37), and cyst wall by salmonE anti-CST1 antibody (1:1,000) (7), anti-MCP4 mouse monoclonal antibody (1:500), or anti-MAG1 mouse monoclonal antibody (1:500). Alexa Fluor 594-conjugated anti-rat, CF405M-conjugated anti-mouse or anti-rabbit or Alexa Fluor 488-conjugated anti-mouse antibodies were used as secondary antibodies (1:1,000). For dense granule staining, extracellular parasites were fixed with 4% PFA and permeabilized with 0.1% Triton X-100 before incubation with anti-HA rat monoclonal antibody 3F10 (1:250) and anti-GRA1 mouse monoclonal antibody 92.10B (1:500) (38). Mounted coverslips were imaged using a Leica TCS SP5 confocal microscope.

### Plaque assay.

Parasites were lysed from host cells by 27-gauge needle and filtered through a 5-μm filter to remove host cell debris. Parasite numbers were counted on a hemocytometer, and 50 parasites of the respective strains were added to triplicate wells containing confluent HFFs in six-well dishes. Parasites were grown for 14 days before fixing and staining with a 20% methanol–0.5% crystal violet solution. Plaque size was determined using ImageJ.

### Morphological electron microscopy.

For ultrastructural analyses, CST3-KO parasites were injected into BALB/c dm1^−/−^ mice, followed by cyst harvesting and purification 3 weeks postinfection, as described previously (7). CST2-HA, CST2-KO, and CST2-COMP cysts were prepared *in vitro* by growth in bradyzoite-inducing conditions for 7 days. Cysts were fixed with 2.5% glutaraldehyde and 2% paraformaldehyde in 0.1 M sodium cacodylate buffer, postfixed with 1% osmium tetroxide followed by 2% uranyl acetate, dehydrated through a graded series of ethanol and embedded in LX112 resin (LADD Research Industries, Burlington, VT). Ultrathin sections were cut on a Leica Ultracut UC7, stained with uranyl acetate, followed by lead citrate, and viewed on a JEOL 1400EX transmission electron microscope at 80 kV.

### Murine survival assay and quantification of cyst numbers and sizes.

Four- to 8-week-old female C57BL/6 mice (The Jackson Laboratory, Bar Harbor, ME) were infected with 10^3^ or 10^5^ tachyzoites of the appropriate strains intraperitoneally. Mortality was observed daily for 30 days until the mice were sacrificed and the brains were harvested. The brains were homogenized with a Wheaton Potter-Elvehjem tissue grinder with a 100- to 150-μm clearance (ThermoFisher) in PBS, and an aliquot of the homogenate was viewed under a Microphoto-FXA epifluorescence microscope (Nikon) to look for GFP fluorescent cysts. Images of these cysts were analyzed with ImageJ to determine their sizes. ANOVA and Tukey HSD test were used to test for significance in cyst numbers and cyst sizes between strains in R. Survival statistics were analyzed by the R package survminer.

### Ethics statement.

All mouse experiments were conducted according to guidelines from the U.S. Public Health Service Policy on Humane Care and Use of Laboratory Animals. Animals were maintained in an AAALAC-approved facility, and all protocols were approved by the Institutional Care Committee of the Albert Einstein College of Medicine, Bronx, NY (Animal Protocol 20150908; Animal Welfare Assurance no. A3312-01).

## ADDENDUM IN PROOF

While this article was in the proof stage, another study was published that used the names GRA47, GRA48, and GRA49 for newly described hypothetical proteins:

Beraki T, Hu X, Broncel M, Young JC, O'Shaughnessy WJ, Borek D, Treeck M, Reese ML. 2019. Divergent kinase regulates membrane ultrastructure of the *Toxoplasma* parasitophorous vacuole. Proc Natl Acad Sci U S A 116:6361–6370. https://doi.org/10.1073/pnas.1816161116.

To avoid confusion, we propose updating the GRA numbering for the hypothetical proteins characterized in this study as follows:

TGME49_203600 – CST2 (GRA50)
TGME49_230705 – CST3 (GRA51)
TGME49_319340 – CST5 (GRA52)
TGME49_260520 – CST6 (GRA53)

We have left comments on the ToxoDB gene pages for the gene IDs listed above regarding this updated nomenclature.

## References

[1] Pappas G, Roussos N, Falagas ME 2009 Toxoplasmosis snapshots: global status of Toxoplasma gondii seroprevalence and implications for pregnancy and congenital toxoplasmosis. Int J Parasitol 39:1385–1394. doi:10.1016/j.ijpara.2009.04.003. 19433092

[2] Sakikawa M, Noda S, Hanaoka M, Nakayama H, Hojo S, Kakinoki S, Nakata M, Yasuda T, Ikenoue T, Kojima T 2012 Anti-Toxoplasma antibody prevalence, primary infection rate, and risk factors in a study of toxoplasmosis in 4,466 pregnant women in Japan. Clin Vaccine Immunol 19:365–367. doi:10.1128/CVI.05486-11. 22205659 PMC3294603

[3] Montoya JG, Liesenfeld O 2004 Toxoplasmosis. Lancet 363:1965–1976. doi:10.1016/S0140-6736(04)16412-X. 15194258

[4] Werner H, Matuschka FR, Brandenburg I 1979 Structural changes of Toxoplasma gondii bradyzoites and cysts following therapy with sulfamethoxypyrazine-pyrimethamine: studies by light and electron microscopy. Consequences for chemotherapy. Zentralbl Bakteriol Orig A 245:240–253. 44617

[5] Torpier G, Charif H, Darcy F, Liu J, Darde ML, Capron A 1993 Toxoplasma gondii: differential location of antigens secreted from encysted bradyzoites. Exp Parasitol 77:13–22. doi:10.1006/expr.1993.1056. 8344403

[6] Buchholz KR, Fritz HM, Chen X, Durbin-Johnson B, Rocke DM, Ferguson DJ, Conrad PA, Boothroyd JC 2011 Identification of tissue cyst wall components by transcriptome analysis of in vivo and in vitro Toxoplasma gondii bradyzoites. Eukaryot Cell 10:1637–1647. doi:10.1128/EC.05182-11. 22021236 PMC3232729

[7] Tomita T, Bzik DJ, Ma YF, Fox BA, Markillie LM, Taylor RC, Kim K, Weiss LM 2013 The Toxoplasma gondii cyst wall protein CST1 is critical for cyst wall integrity and promotes bradyzoite persistence. PLoS Pathog 9:e1003823. doi:10.1371/journal.ppat.1003823. 24385904 PMC3873430

[8] Buchholz KR, Bowyer PW, Boothroyd JC 2013 Bradyzoite pseudokinase 1 is crucial for efficient oral infectivity of the Toxoplasma gondii tissue cyst. Eukaryot Cell 12:399–410. doi:10.1128/EC.00343-12. 23291621 PMC3629768

[9] Zhang YW, Halonen SK, Ma YF, Tanowtiz HB, Weiss LM 2010 A purification method for enrichment of the Toxoplasma gondii cyst wall. J Neuroparasitology 1:N101001. doi:10.4303/jnp/N101001. 21687827 PMC3115730

[10] Sinai AP, Webster P, Joiner KA 1997 Association of host cell endoplasmic reticulum and mitochondria with the Toxoplasma gondii parasitophorous vacuole membrane: a high affinity interaction. J Cell Sci 110:2117–2128. 9378762 10.1242/jcs.110.17.2117

[11] Satori CP, Kostal V, Arriaga EA 2012 Review on recent advances in the analysis of isolated organelles. Anal Chim Acta 753:8–18. doi:10.1016/j.aca.2012.09.041. 23107131 PMC3484375

[12] Nadipuram SM, Kim EW, Vashisht AA, Lin AH, Bell HN, Coppens I, Wohlschlegel JA, Bradley PJ 2016 In vivo biotinylation of the Toxoplasma parasitophorous vacuole reveals novel dense granule proteins important for parasite growth and pathogenesis. mBio 7:e00808-16. doi:10.1128/mBio.00808-16. 27486190 PMC4981711

[13] Pan M, Li M, Li L, Song Y, Hou L, Zhao J, Shen B 2018 Identification of novel dense-granule proteins in Toxoplasma gondii by two proximity-based biotinylation approaches. J Proteome Res 18:319–330. 30362762 10.1021/acs.jproteome.8b00626

[14] Friedrich N, Santos JM, Liu Y, Palma AS, Leon E, Saouros S, Kiso M, Blackman MJ, Matthews S, Feizi T, Soldati-Favre D 2010 Members of a novel protein family containing microneme adhesive repeat domains act as sialic acid-binding lectins during host cell invasion by apicomplexan parasites. J Biol Chem 285:2064–2076. doi:10.1074/jbc.M109.060988. 19901027 PMC2804363

[15] Coffey MJ, Dagley LF, Seizova S, Kapp EA, Infusini G, Roos DS, Boddey JA, Webb AI, Tonkin CJ 2018 Aspartyl protease 5 matures dense granule proteins that reside at the host-parasite interface in Toxoplasma gondii. mBio 9:e01796-18. doi:10.1128/mBio.01796-18. 30377279 PMC6212819

[16] Sugi T, Kato K, Weiss LM 2016 An improved method for introducing site-directed point mutation into the Toxoplasma gondii genome using CRISPR/Cas9. Parasitol Int 65:558–562. doi:10.1016/j.parint.2016.05.002. 27167504 PMC5035577

[17] Shen B, Brown K, Long S, Sibley LD 2017 Development of CRISPR/Cas9 for efficient genome editing in Toxoplasma gondii. Methods Mol Biol 1498:79–103. doi:10.1007/978-1-4939-6472-7_6. 27709570

[18] Shastri AJ, Marino ND, Franco M, Lodoen MB, Boothroyd JC 2014 GRA25 is a novel virulence factor of Toxoplasma gondii and influences the host immune response. Infect Immun 82:2595–2605. doi:10.1128/IAI.01339-13. 24711568 PMC4019154

[19] Adomako-Ankomah Y, English ED, Danielson JJ, Pernas LF, Parker ML, Boulanger MJ, Dubey JP, Boyle JP 2016 Host mitochondrial association evolved in the human parasite Toxoplasma gondii via neofunctionalization of a gene duplicate. Genetics 203:283–298. doi:10.1534/genetics.115.186270. 26920761 PMC4858780

[20] Franco M, Panas MW, Marino ND, Lee MC, Buchholz KR, Kelly FD, Bednarski JJ, Sleckman BP, Pourmand N, Boothroyd JC 2016 A novel secreted protein, MYR1, is central to Toxoplasma’s manipulation of host cells. mBio 7:e02231-15. 26838724 10.1128/mBio.02231-15 PMC4742717

[21] Lorestani A, Ivey FD, Thirugnanam S, Busby MA, Marth GT, Cheeseman IM, Gubbels MJ 2012 Targeted proteomic dissection of Toxoplasma cytoskeleton sub-compartments using MORN1. Cytoskeleton (Hoboken) 69:1069–1085. doi:10.1002/cm.21077. 23027733 PMC3566231

[22] Limper AH, Weiss LM 2011 Guidelines for the naming of genes, gene products, and mutants in the opportunistic protists. J Eukaryot Microbiol 58:537–538. doi:10.1111/j.1550-7408.2011.00577.x. 21883634 PMC3703751

[23] Sibley LD, Pfefferkorn ER, Boothroyd JC 1991 Proposal for a uniform genetic nomenclature in Toxoplasma gondii. Parasitol Today 7:327–328. doi:10.1016/0169-4758(91)90210-F. 15463406

[24] Gold DA, Kaplan AD, Lis A, Bett GC, Rosowski EE, Cirelli KM, Bougdour A, Sidik SM, Beck JR, Lourido S, Egea PF, Bradley PJ, Hakimi MA, Rasmusson RL, Saeij JP 2015 The Toxoplasma dense granule proteins GRA17 and GRA23 mediate the movement of small molecules between the host and the parasitophorous vacuole. Cell Host Microbe 17:642–652. doi:10.1016/j.chom.2015.04.003. 25974303 PMC4435723

[25] Lemgruber L, Lupetti P, Martins-Duarte ES, De Souza W, Vommaro RC 2011 The organization of the wall filaments and characterization of the matrix structures of Toxoplasma gondii cyst form. Cell Microbiol 13:1920–1932. doi:10.1111/j.1462-5822.2011.01681.x. 21899696

[26] Bougdour A, Durandau E, Brenier-Pinchart MP, Ortet P, Barakat M, Kieffer S, Curt-Varesano A, Curt-Bertini RL, Bastien O, Coute Y, Pelloux H, Hakimi MA 2013 Host cell subversion by Toxoplasma GRA16, an exported dense granule protein that targets the host cell nucleus and alters gene expression. Cell Host Microbe 13:489–500. doi:10.1016/j.chom.2013.03.002. 23601110

[27] Braun L, Brenier-Pinchart MP, Yogavel M, Curt-Varesano A, Curt-Bertini RL, Hussain T, Kieffer-Jaquinod S, Coute Y, Pelloux H, Tardieux I, Sharma A, Belrhali H, Bougdour A, Hakimi MA 2013 A Toxoplasma dense granule protein, GRA24, modulates the early immune response to infection by promoting a direct and sustained host p38 MAPK activation. J Exp Med 210:2071–2086. doi:10.1084/jem.20130103. 24043761 PMC3782045

[28] Krishnamurthy S, Saeij JPJ 2018 Toxoplasma does not secrete the GRA16 and GRA24 effectors beyond the parasitophorous vacuole membrane of tissue cysts. Front Cell Infect Microbiol 8:366. doi:10.3389/fcimb.2018.00366. 30406043 PMC6201044

[29] Curt-Varesano A, Braun L, Ranquet C, Hakimi MA, Bougdour A 2016 The aspartyl protease TgASP5 mediates the export of the Toxoplasma GRA16 and GRA24 effectors into host cells. Cell Microbiol 18:151–167. doi:10.1111/cmi.12498. 26270241

[30] Coffey MJ, Sleebs BE, Uboldi AD, Garnham A, Franco M, Marino ND, Panas MW, Ferguson DJ, Enciso M, O’Neill MT, Lopaticki S, Stewart RJ, Dewson G, Smyth GK, Smith BJ, Masters SL, Boothroyd JC, Boddey JA, Tonkin CJ 2015 An aspartyl protease defines a novel pathway for export of Toxoplasma proteins into the host cell. Elife 4:e10809. doi:10.7554/eLife.10809. 26576949 PMC4764566

[31] Hammoudi PM, Jacot D, Mueller C, Di Cristina M, Dogga SK, Marq JB, Romano J, Tosetti N, Dubrot J, Emre Y, Lunghi M, Coppens I, Yamamoto M, Sojka D, Pino P, Soldati-Favre D 2015 Fundamental roles of the Golgi-associated Toxoplasma aspartyl protease, ASP5, at the host-parasite interface. PLoS Pathog 11:e1005211. doi:10.1371/journal.ppat.1005211. 26473595 PMC4608785

[32] Pernas L, Adomako-Ankomah Y, Shastri AJ, Ewald SE, Treeck M, Boyle JP, Boothroyd JC 2014 Toxoplasma effector MAF1 mediates recruitment of host mitochondria and impacts the host response. PLoS Biol 12:e1001845. doi:10.1371/journal.pbio.1001845. 24781109 PMC4004538

[33] Milligan-Myhre K, Wilson SK, Knoll LJ 2016 Developmental change in translation initiation alters the localization of a common microbial protein necessary for Toxoplasma chronic infection. Mol Microbiol 102:1086–1098. doi:10.1111/mmi.13538. 27671212 PMC5161674

[34] Chen AL, Kim EW, Toh JY, Vashisht AA, Rashoff AQ, Van C, Huang AS, Moon AS, Bell HN, Bentolila LA, Wohlschlegel JA, Bradley PJ 2015 Novel components of the Toxoplasma inner membrane complex revealed by BioID. mBio 6:e02357-14. 25691595 10.1128/mBio.02357-14 PMC4337574

[35] Sugi T, Ma YF, Tomita T, Murakoshi F, Eaton MS, Yakubu R, Han B, Tu V, Kato K, Kawazu S, Gupta N, Suvorova ES, White MW, Kim K, Weiss LM 2016 Toxoplasma gondii cyclic AMP-dependent protein kinase subunit 3 is involved in the switch from tachyzoite to bradyzoite development. mBio 7:e00755-16. doi:10.1128/mBio.00755-16. 27247232 PMC4895117

[36] Tomita T, Sugi T, Yakubu R, Tu V, Ma Y, Weiss LM 2017 Making home sweet and sturdy: Toxoplasma gondii ppGalNAc-Ts glycosylate in hierarchical order and confer cyst wall rigidity. mBio 8:e02048-16. doi:10.1128/mBio.02048-16. 28074022 PMC5225312

[37] Sugi T, Kato K, Kobayashi K, Watanabe S, Kurokawa H, Gong H, Pandey K, Takemae H, Akashi H 2010 Use of the kinase inhibitor analog 1NM-PP1 reveals a role for Toxoplasma gondii CDPK1 in the invasion step. Eukaryot Cell 9:667–670. doi:10.1128/EC.00351-09. 20173034 PMC2863409

[38] Weiss LM, Laplace D, Takvorian PM, Tanowitz HB, Cali A, Wittner M 1995 A cell culture system for study of the development of Toxoplasma gondii bradyzoites. J Eukaryot Microbiol 42:150–157. doi:10.1111/j.1550-7408.1995.tb01556.x. 7757057

