# Supplementary figures and images for: Enrichment and Proteomic Characterization of the Cyst Wall from In Vitro Toxoplasma gondii Cysts
Source: mBio. 2019 Apr 30;10(2):e00469-19. doi: 10.1128/mBio.00469-19 (PMC6495374; doi:10.1128/mBio.00469-19)

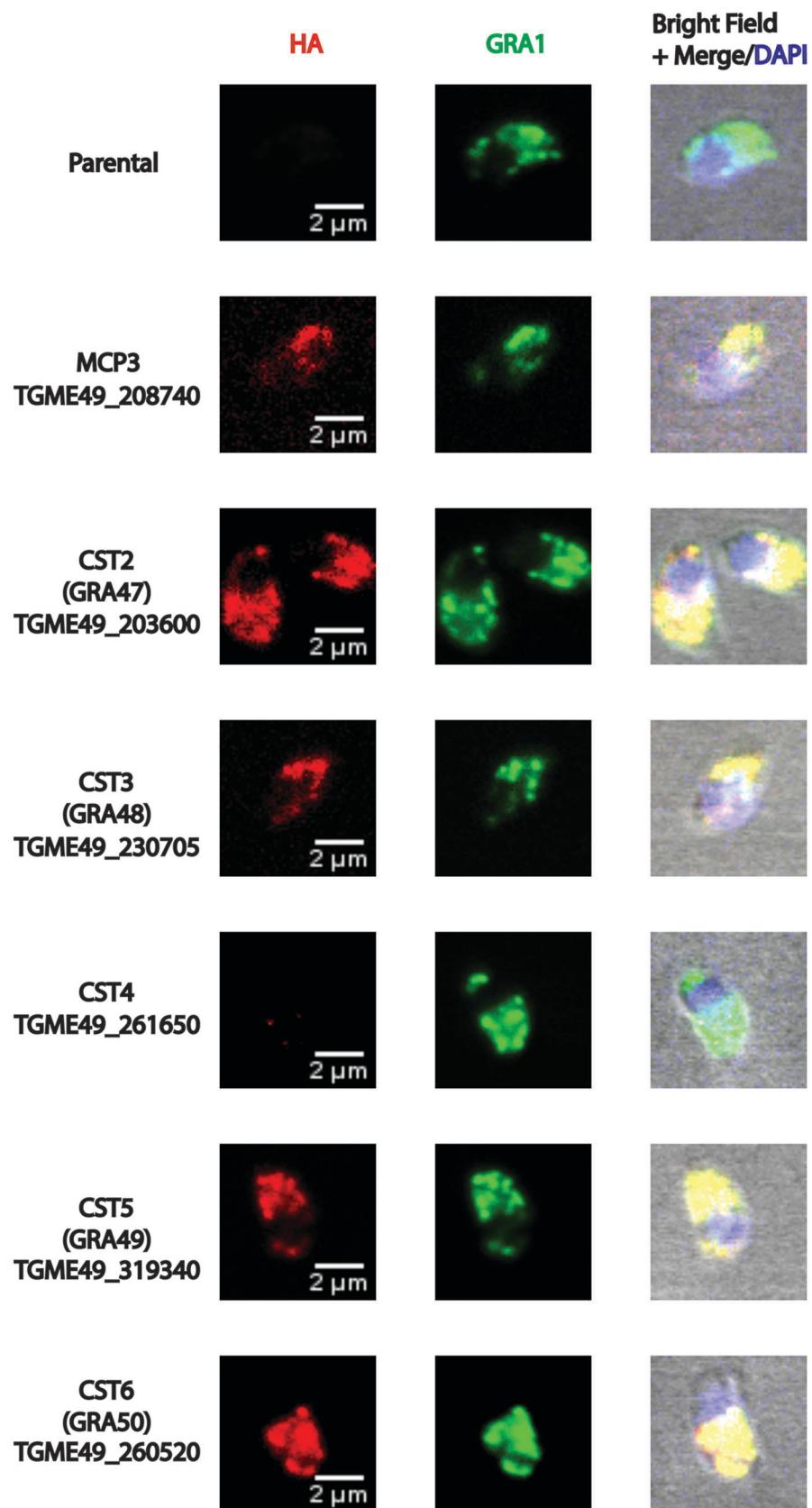

Supplement: FIG S1 [file mBio.00469-19-sf001.pdf]

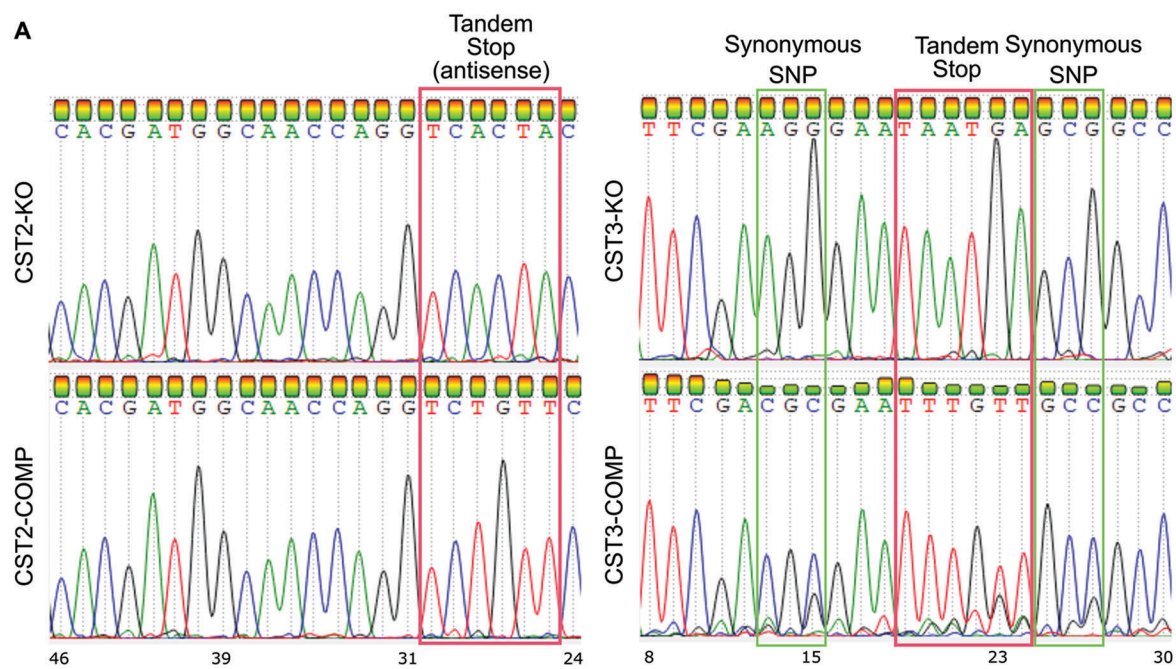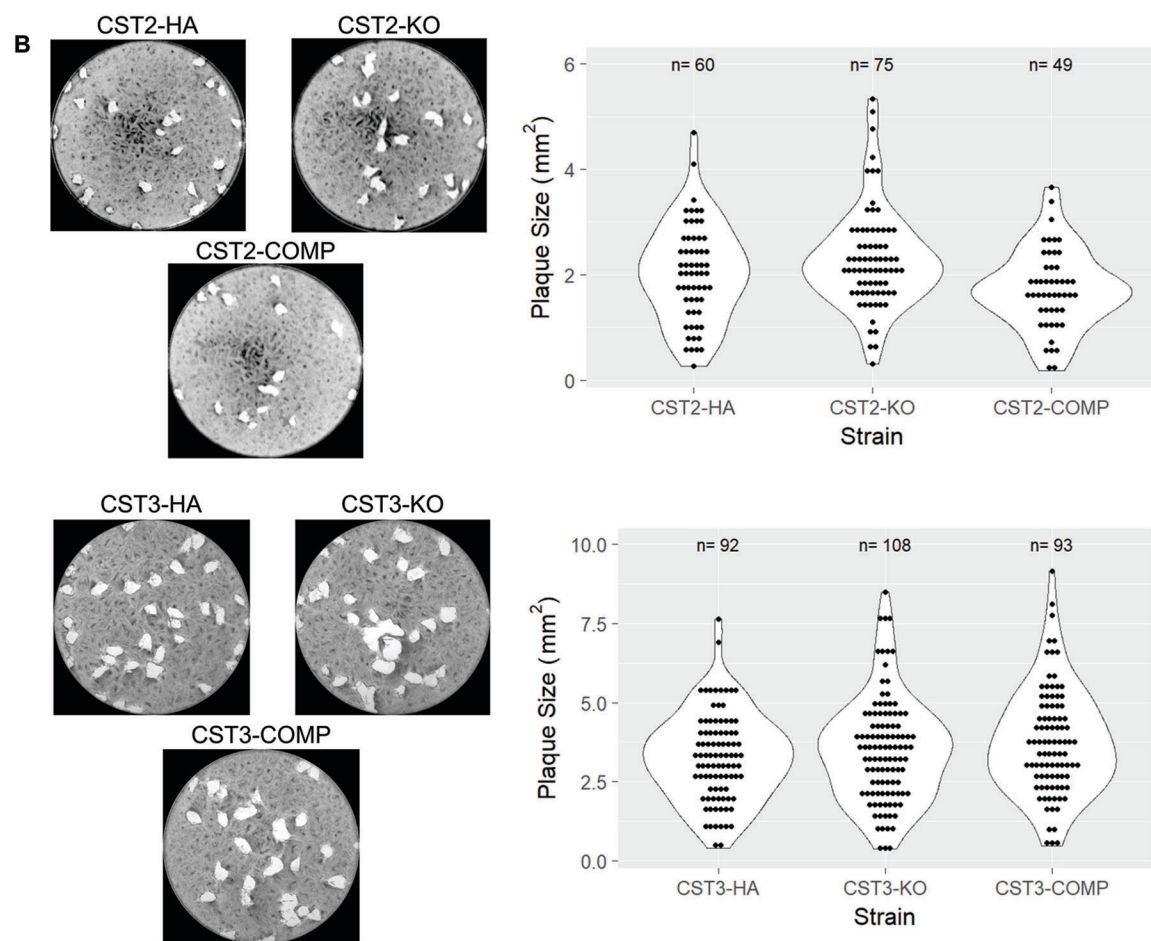

Supplement: FIG S2 [file mBio.00469-19-sf002.pdf]

**A**

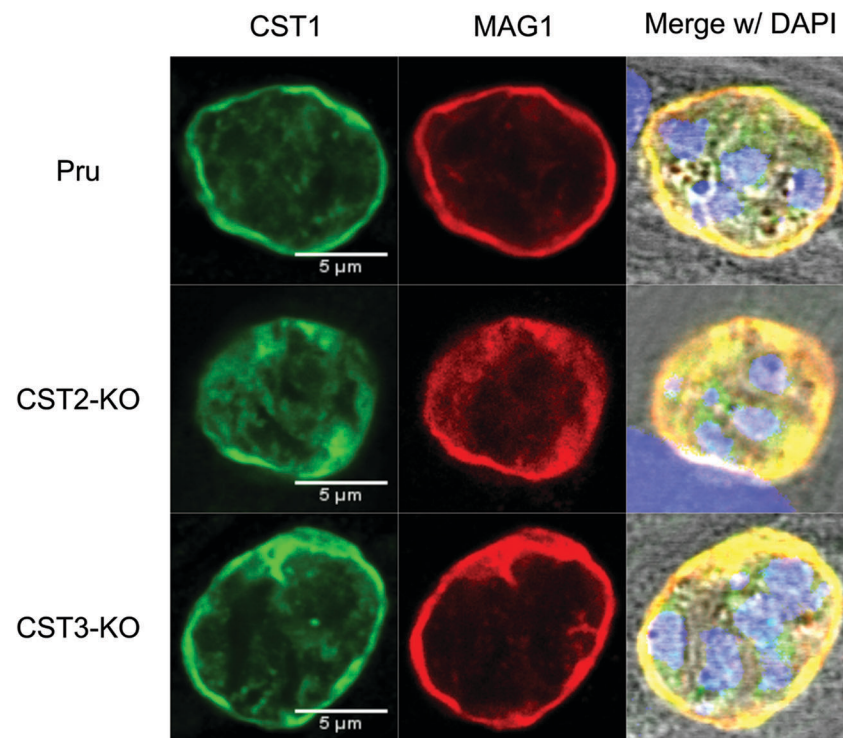

**B**

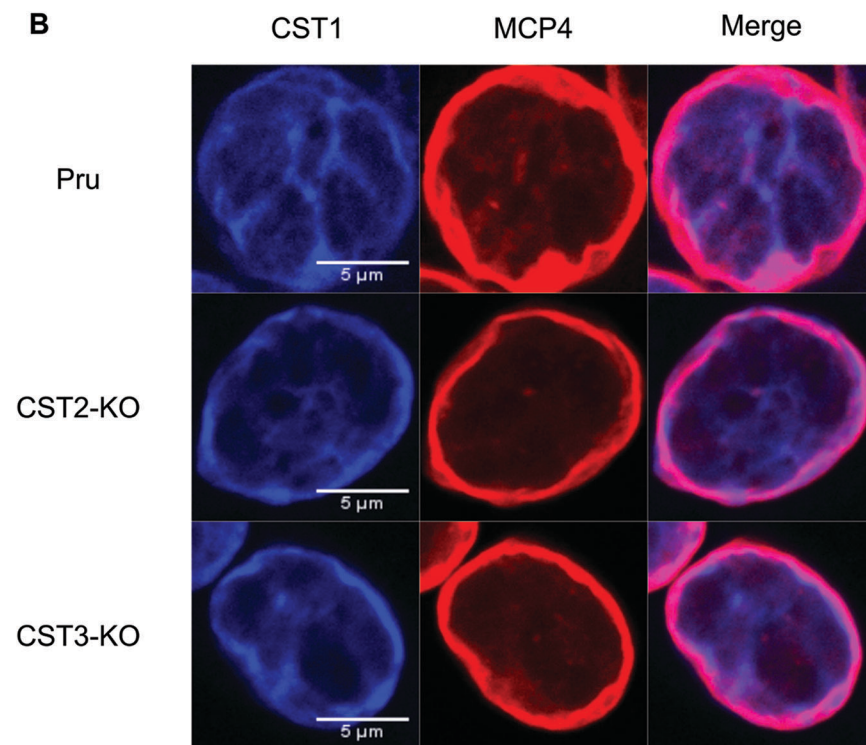

Supplement: FIG S3 [file mBio.00469-19-sf003.pdf]

**A**

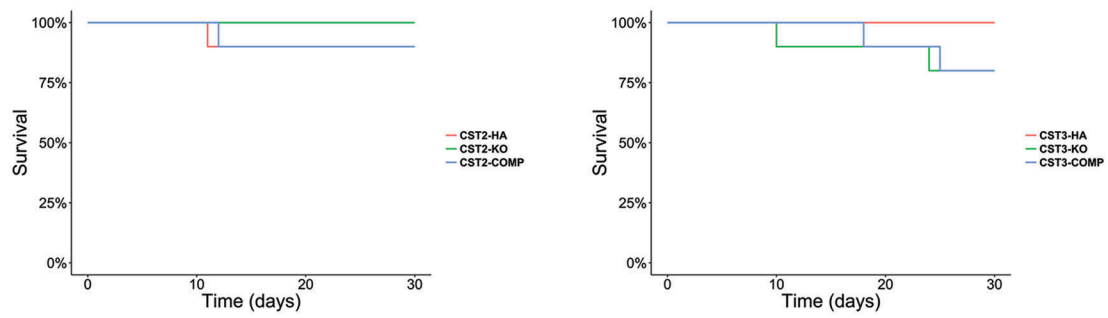

**B**

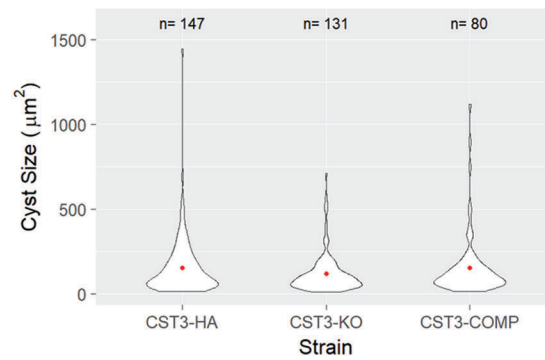

**C**

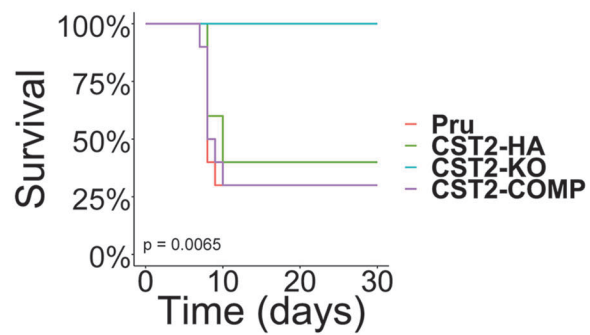

Supplement: FIG S4 [file mBio.00469-19-sf004.pdf]
