# Supplementary material for: Enrichment and Proteomic Characterization of the Cyst Wall from In Vitro Toxoplasma gondii Cysts
Source: mBio. 2019 Apr 30;10(2):e00469-19. doi: 10.1128/mBio.00469-19 (PMC6495374; doi:10.1128/mBio.00469-19)
Supplement: TABLE S1 [file mBio.00469-19-st001.docx]

**Table S1: Oligomers used as donor DNA**

| Name | Sequence |
| --- | --- |
| CST2_C-term_1xHA_FWD | GATTGCTGCTGCGATAATAAGGCATACCCTTACGATGTACCGGATTACGCATaAGATAAGTAGATcGCTAATGCCGCGAAGCAACcGAAACGAACGGACA |
| CST2_C-term_1xHA_RVS | TGTCCGTTCGTTTCgGTTGCTTCGCGGCATTAGCgATCTACTTATCTtATGCGTAATCCGGTACATCGTAAGGGTATGCCTTATTATCGCAGCAGCAATC |
| CST3_C-term_1xHA_FWD | ATTCGTTAACTCTTTCCCGGAGTCGCGTGAACCGAAATACCCTTACGATGTACCGGATTACGCATaAAGGTTTTGTGGTTCGAGAACGAGCGGTCGAAGT |
| CST3_C-term_1xHA_RVS | ACTTCGACCGCTCGTTCTCGAACCACAAAACCTTTATGCGTAATCCGGTACATCGTAAGGGTATTTCGGTTCACGCGACTCCGGGAAAGAGTTAACGAAT |
| CST2_KO_FWD | TGACGACTTCAACGCTCTTGCACCGTTTTCCCGGGATGATTCTCTCCACGGAAAGAATGtagtgaCCTGGTTGCCATCGTGCCTCGCGAAATGGCGGAAG |
| CST2_KO_RVS | CTTCCGCCATTTCGCGAGGCACGATGGCAACCAGGtcactaCATTCTTTCCGTGGAGAGAATCATCCCGGGAAAACGGTGCAAGAGCGTTGAAGTCGTCA |
| CST3_KO_FWD | GTGTGCTGTTCGGATACTTCACGGGTAGAAAAGCCATGGTTATTCGAAGGGAAtaatgaGCGGCCGTCGCTGCATTCGCACTCCTCCCCCTACTTATTGC |
| CST3_KO_RVS | GCAATAAGTAGGGGGAGGAGTGCGAATGCAGCGACGGCCGCtcattaTTCCCTTCGAATAACCATGGCTTTTCTACCCGTGAAGTATCCGAACAGCACAC |
| CST2_COMP_FWD | TGACGACTTCAACGCTCTTGCACCGTTTTCCCGGGATGATTCTCTCCaccGAAAGAATGaacagaCCTGGTTGCCATCGTGCCTCGCGAAATGGCGGAAG |
| CST2_COMP_RVS | CTTCCGCCATTTCGCGAGGCACGATGGCAACCAGGtctgttCATTCTTTCggtGGAGAGAATCATCCCGGGAAAACGGTGCAAGAGCGTTGAAGTCGTCA |
| CST3_COMP_FWD | GTGTGCTGTTCGGATACTTCACGGGTAGAAAAGCCATGGTTATTCGAcgcGAATTTGTTgccGCCGTCGCTGCATTCGCACTCCTCCCCCTACTTATTGC |
| CST3_COMP_RVS | GCAATAAGTAGGGGGAGGAGTGCGAATGCAGCGACGGCggcAACAAATTCgcgTCGAATAACCATGGCTTTTCTACCCGTGAAGTATCCGAACAGCACAC |

Mutations are designated in red
