# Supplementary material for: Enrichment and Proteomic Characterization of the Cyst Wall from In Vitro Toxoplasma gondii Cysts
Source: mBio. 2019 Apr 30;10(2):e00469-19. doi: 10.1128/mBio.00469-19 (PMC6495374; doi:10.1128/mBio.00469-19)
Supplement: TABLE S2 [file mBio.00469-19-st002.docx]

| Name | Sequence | Purpose |
| --- | --- | --- |
| MCP3_3xHA-TAG_FWD | ATTACGCCAAGCTCGGAACAATGGTGTGGAGAACGGGA | C-terminal tagging |
| MCP3_3xHA-TAG_RVS | GACGTCGTACGGGTACCTGTATCCCCGTAGCCTTCGAT | C-terminal tagging |
| CST2_3xHA-TAG_FWD | ATTACGCCAAGCTCGGAAACGTGCTACCACGAAACAGA | C-terminal tagging |
| CST2_3xHA-TAG_RVS | GACGTCGTACGGGTACCTTGCCTTATTATCGCAGCAGC | C-terminal tagging |
| CST3_3xHA-TAG_FWD | ATTACGCCAAGCTCGGAAGACGCATGAGGAGAGGAAAG | C-terminal tagging |
| CST3_3xHA-TAG_RVS | GACGTCGTACGGGTACCTTTTCGGTTCACGCGACTC | C-terminal tagging |
| CST4_3xHA-TAG_FWD | ATTACGCCAAGCTCGGAATCGACTTCTGGTTGGGAAGC | C-terminal tagging |
| CST4_3xHA-TAG_RVS | GACGTCGTACGGGTACCTgtcctcctcagcgataaaaaag | C-terminal tagging |
| CST5_3xHA-TAG_FWD | ATTACGCCAAGCTCGGAAGACCGCAAGGAAGTCTGACA | C-terminal tagging |
| CST5_3xHA-TAG_RVS | GACGTCGTACGGGTACCTgtacgagttgagcaagtcacga | C-terminal tagging |
| CST6_3xHA-TAG_FWD | ATTACGCCAAGCTCGGAATCAGAAGCAAGCTGCGGTAA | C-terminal tagging |
| CST6_3xHA-TAG_RVS | GACGTCGTACGGGTACCTgtcaaaatcgtcgttcagatcg | C-terminal tagging |
| sgCST2_C-term | GTAAGGCATGAGATAAGTAGAGTTTTAGAGCTAGAAATAG | Endogenous C-terminal tagging |
| sgCST3_C-term | GAACCACAAAACCTTCATTTGTTTTAGAGCTAGAAATAGC | Endogenous C-terminal tagging |
| sgCST2_KO | GACGGAAAGAATGAATCGCCCGTTTTAGAGCTAGAAATAG | Knocking out CST2 |
| sgCST3_KO | GTATTCGAAGGGAATTTGTTGGTTTTAGAGCTAGAAATAG | Knocking out CST3 |
| sgCST2_COMP | GTCCCGGGATGATTCTCTCCAGTTTTAGAGCTAGAAATAG | Complementing CST2-KO |
| sgCST3_COMP | GAAAAGCCATGGTTATTCGAGTTTTAGAGCTAGAAATAGC | Complementing CST3-KO |

**Table S2: Primers used for cloning or sequencing**
